# Achieving the endgame: Integrated NTD case searches

**DOI:** 10.1371/journal.pntd.0006623

**Published:** 2018-12-20

**Authors:** Lucas Buyon, Randall Slaven, Paul M. Emerson, Jonathan King, Oscar Debrah, Agatha Aboe, Ernesto Ruiz-Tiben, E. Kelly Callahan

**Affiliations:** 1 Harvard T.H. Chan School of Public Health, Department of Immunology and Infectious Diseases, Boston, Massachusetts, United States of America; 2 The Carter Center, Atlanta, Georgia, United States of America; 3 International Trachoma Initiative, Decatur, Georgia, United States of America; 4 World Health Organization, Geneva, Switzerland; 5 Eye Care Unit, Ghanaian Health Service, Accra, Ghana; 6 Sightsavers International, Boston, Massachusetts, United States of America; RTI International, UNITED REPUBLIC OF TANZANIA

## Abstract

Trachoma and Guinea Worm Disease (GWD) are neglected tropical diseases (NTD) slated for elimination as a public health problem and eradication respectively by the World Health Organization. As these programs wind down, uncovering the last cases becomes an urgent priority. In 2010, Ghana Health Services, along with The Carter Center, Sightsavers, and other partners, conducted integrated case searches for both GWD and the last stage of trachoma disease, trachomatous trichiasis (TT), as well as providing surgical treatment for TT to meet elimination (and eradication targets). House to house case searches for both diseases were conducted and two case management strategies were explored: a centralized referral to services method and a Point of Care (POC) delivery method. 835 suspected TT cases were discovered in the centralized method, of which 554 accepted surgery. 482 suspected TT cases were discovered in the POC method and all TT cases accepted surgery. The cost per TT case examined was lower in the POC searches compared to the centralized searches ($19.97 in the POC searches and $20.85 in the centralized searches). Both strategies resulted in high surgical uptake for TT surgery, with average uptakes of 72.4% and 83.9% for the centralized and POC searches respectively. We present here that house to house case searches offering services at POC are feasible and a potential tool for elimination and eradication programs nearing their end.

## Author summary

Trachoma and Guinea Worm Disease (GWD) are neglected tropical diseases slated for elimination as a public health problem and eradication respectively by the World Health Organization. As these programs wind down, uncovering the last cases becomes an urgent priority in order to confirm that eradiation and elimination targets have been reached. Active case searches are one method of finding these last vestiges of disease. Searches that look for multiple diseases are referred to as integrated searches. We piloted here integrated house-to-house case searches for GWD and Trachoma with two case management strategies; a referral approach to a central location and a point of care approach (POC)–offering services at the household or ‘point of care’ location. POC approaches can be difficult to implement in low resource settings because they require extensive personnel, financial commitment, and logistical support. However, POC approaches remove one of the biggest barriers to treatment, time spent traveling to a health center, and thus can improve treatment acceptance. We found here that integrated active case searches with a POC case management approach can be implemented in a low resource setting; and improve acceptance and uptake of trachoma examination and trichiasis surgery respectively, without costing much more than a referral-based case management approach.

## Introduction

The neglected tropical diseases (NTDs) are a diverse group of diseases that affect the poorest of the poor, primarily in rural Africa, Asia and Latin America.[1] Eight NTDs are slated for elimination, and two: yaws and *Dracunculus medinensis*, or Guinea worm disease (GWD), are targeted for eradication by World Health Organization (WHO) resolutions WHA39. [2] 1and WHA 66.12. [3] If eradication is successful, they will join smallpox and rinderpest as the only diseases eradicated from the world. Trachoma is targeted for elimination as a public health problem by the year 2020 by the WHO resolution WHA 51.11. [4]

Guinea worm disease is a parasitic disease. Individuals become infected by drinking from a stagnant water source that contains copepods which carry larva from the parasitic nematode roundworm *Dracunculiasis medinensis*. [5] Once ingested, *D*. *medinensis* larva mature and mate inside the host’s body. Approximately one year later, a female worm will emerge through a blister, causing immense pain. Infected persons often seek relief from the burning sensation by immersing the wound in a water source, at which point the worm releases its larvae.[5]There is no known medical treatment available to kill the parasitic nematode while it is inside the host, and on average GWD incapacitates its victims for about 8.5 weeks [5,6] Eradication efforts for GWD primarily use behavioral and environmental interventions to break the cycle of transmission to prevent contamination of drinking water sources and by improving access to clean drinking water [7] The eradication of GWD requires that every case must be found and contained, making surveillance and case finding a priority for countries on the verge of elimination.

Trachoma is caused by a bacterium, *Chlamydia trachomatis*, and is spread via direct contact with ocular and nasal discharge from an infected person, including unwashed towels or clothes, as well as eye-seeking flies. [8] Over time, repeated cycles of infection and resolution result in scar formation on the inner eyelid, which contract and physically distort the eyelid. Due to this scarring and distortion, the eyelashes turn inward and begin to scrape the globe of the eye, causing trauma to the cornea that can result in blindness. [9]This stage is referred to as trachomatous trichiasis (TT). Control for trachoma utilizes the WHO endorsed SAFE strategy (surgery, antibiotics, facial cleanliness, and environmental improvement). [10] Trachomatous trichiasis can be addressed via surgery, which consists of a simple eye-lid rotation to stop the lashes from scratching the globe of the eye. Surgery to reduce the prevalence of TT in a district is critical to achieving one of the targets set by WHO for elimination of Trachoma as a public health problem (a prevalence of TT of < 1 case per 1000 total population). [11] This makes detecting and treating cases of TT a priority for trachoma programs as they reach elimination thresholds.

For countries in the end stage for both eradication of GWD and elimination of trachoma as a public health problem, or elimination of other NTDs, there exists an opportunity for integrating case searches for the diseases of interest. As GWD eradication and national efforts to eliminate trachoma draw closer to their elimination thresholds, detecting the remaining cases is paramount. Ghana is one such country that reached this stage of both eradication of GWD and trachoma elimination in early 2010. Prior to the start of the trachoma elimination campaign, the Ghana Health Services (GHS), the implementing agency of the Ministry of Health, had set a goal of treating all unaddressed cases of TT. This aggressive goal exceeded the elimination threshold (1 TT case/1000 persons) set by WHO, but would ensure that Ghana would meet the requirements of elimination. [11–13]In 2010 the GHS, assisted by The Carter Center and Sightsavers, collaborated to conduct house-to-house case searches to identify the remaining TT cases to help meet this threshold. Concurrently, Ghana was in the certification of elimination process for GWD, presenting an opportunity to develop integrated case searches for both diseases. Because the TT case searches were already being conducted, the GHS, The Carter Center, and Sightsavers, realized that it would be feasible and likely cost efficient to pilot a program to integrate rumor investigation for GWD in these existing searches. This created the opportunity to determine if an active case search was a feasible strategy, and in this instance, to jointly uncover cases of TT and investigate rumors of GWD to help aid in the goal of elimination and eradication of these diseases.

House-to-house case searches for TT and GWD had not been previously integrated by the GHS. This type of search would ideally provide a fine-scale picture of the disease burden in previously endemic areas because they are conducted on the household level. However, such searches are labor intensive and can be difficult to implement. Therefore, this presented an opportunity to assess the feasibility of integrated house-to-house case searches. Furthermore, since uptake of services to examine and treat TT are often low [13–15], mostly due to logistical barriers and the economic burden resulting from these logistical barriers, the integrated case searches were coupled with either a centralized case management approach (where patients were referred to a central care facility) or point of care case management approach (POC, where patients were offered care either within or near their home). This would enable assessment of: 1) the capacity to integrate case finding for multiple diseases and 2) the impact of two case management strategies on TT examination and surgical acceptance or uptake: centralized case management versus POC case management.

## Methods

In 2009, Ghana Health Services, through surveys, identified 29 districts with a TT backlog. Of the 29 districts, fourteen were in the Northern Region of the country with six of these previously known to be endemic for GWD. In an effort to ensure these six districts were free from GWD and all rumors of the disease investigated and to assist in clearing the TT backlog as efficiently as possible, The Carter Center agreed to assist in integrated cases searches.

The case searches consisted of active house-to-house searching with teams walking from house-to-house to identify cases. Search teams were comprised of GHS staff, Carter Center staff, and village-based health workers (VBHWs). Districts were mapped using existing data prior to the searches to identify villages, regional centers, and household locations, to ensure that no households in each district were not searched. GHS was responsible for the primary ideation and strategy of the case searches, the overall management of the searches, and, in conjunction with The Carter Center, the training of the case search teams. The Carter Center was additionally responsible for the ideation of the integrated case searches, logistics, and supplies for the search teams. Sightsavers was involved with the training of the surgical teams and aids, as well assisting with reporting and financing the searches.

The TT case searches were house-to-house searches, with team members describing to each household a case of TT to persons in the local language and asking if they had seen such presentations. The GWD searches were conducted during the same household visits, with team members showing photos of non-emergent and emergent guinea worm, and asking in the local language if they had seen such presentations. Teams would use chalk to mark households for TT or GWD to verify the case search was completed and the subsequent findings for further investigation.

**Fig 1:**
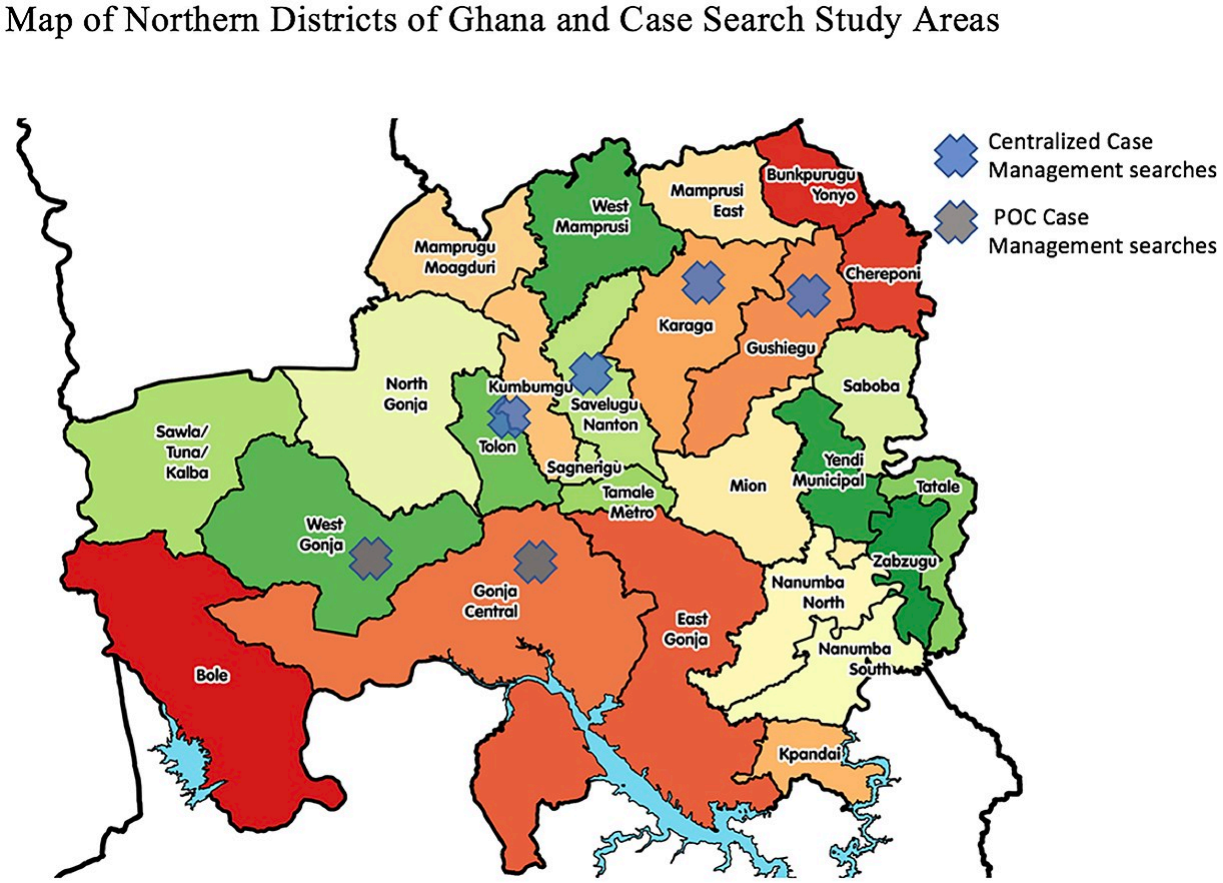
Map of Northern districts of Ghana and case search study areas.

The two searches were different due to a) geographic location (Fig 1) [16] and b) TT case management approaches. The first, conducted in February 2010, utilized a passive community referral system and centralized surgical units, referred to as the centralized case management approach. The second, conducted in November 2010, offered point of care services with surgical services either in homes or within 0.5 km of walking distance (e.g. the center of a village), referred to as the Point of Care (POC) management approach.

Both the centralized case management and POC searches were house-to-house case searches and followed the search strategy outlined above; however, there were two main differences; 1) in the training of search teams and 2) in the TT case management approach. For both the centralized and POC case management approaches, the number of GWD and TT cases investigated and confirmed were recorded, as well as the number of persons eligible for TT surgery, and the number accepting TT surgery. The cost of paying the healthcare workers and surgeons, as well as logistical and surgical costs were calculated by the hours for each professional and the number of surgeries ultimately performed. The costs generated represent the integrated searches, not solely for the cost of TT screening.

### Centralized case management approach

The centralized case management approach was conducted in four districts: Savelugu, Tolon-Kumbungu, Gushegu, and Karaga (Fig 1). Searches were conducted by teams of Guinea worm VBHW. These teams were trained to identify TT cases as a group at a central location the day before the district wide searches began. Training included identifying emergent guinea worm and identify TT cases based on the WHO grading system. A total of 959 health workers staffed this search, and the vast majority of the health workers were male. The searchers were deployed in teams of two to search house-to-house.

The workflow for the search was as follows: search teams moved through mapped areas house to house to enter and search for cases of GWD and TT. All persons exhibiting signs or symptoms consistent with GWD were transported to a case containment center for verification and all persons with suspected TT were referred to a central location for examination and, if eligible given a referral for surgery at that same location. If a surgical team was not nearby, referred patients were told where and when to report for surgery, at an assigned hospital, clinic, or school within 10 kilometers of their village. If a surgical team was nearby, the patients were told to go to that location immediately to receive surgical services as warranted.

### Point of care case management approach

The POC case management approach focused on delivery of TT examination and surgery at the point of care and was conducted in two districts: Central Gonja and West Gonja (Fig 1). Each team had increased numbers of volunteers relative to the centralized searches. In total, 1,833 people were involved in the POC case management approach. The Red Cross volunteer group, were a significant majority of these human resources. Training for these search teams was conducted the day before each localized case search (specifically the day before searching a section of the district) to ensure high recall and preparedness. Training included identifying emergent guinea worm and TT cases based on the WHO grading system. The searchers were deployed in teams of three to search house-to-house. This training strategy contrasted with the centralized case management approach, where all teams where trained simply the day before each districts campaign began.

The workflow for POC case management was as follows: Search teams went house to house to search for cases of GWD and TT. Persons screened to have signs or symptoms consistent with TT were placed on a list of possible cases, and subsequently diagnosed by a healthcare professional in or near their home. As with the centralized case management approach, individuals exhibiting signs or symptoms consistent with GWD were transported to a case containment center for verification. After diagnosis, patients were treated either in their own home or within 0.5 kilometers away if they consented to treatment. The POC case management approach used a mobile surgery team familiar with the local conditions. The surgical teams could perform TT surgery in the house of the TT patient and provide follow up care in the same location.

### Analysis

Basic calculations were conducted using Microsoft Excel. The number of persons reached was estimated by multiplying the number of households reached by the average household size in Ghana (4.5 persons per household). [17]

## Results

**Fig 2:**
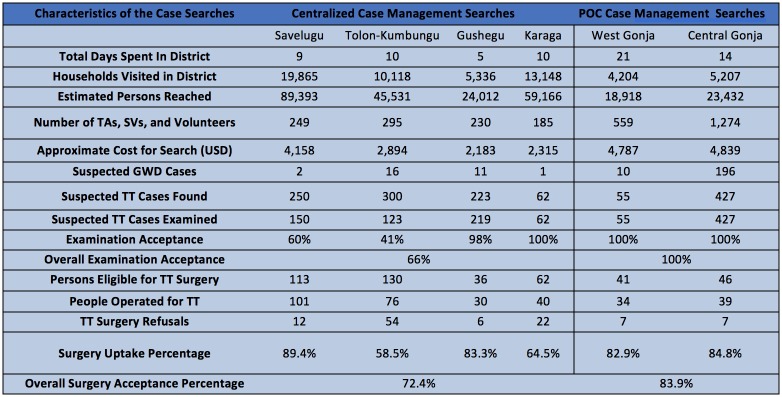
Table of characteristics and outcomes of the case searches.

**Fig 3:**
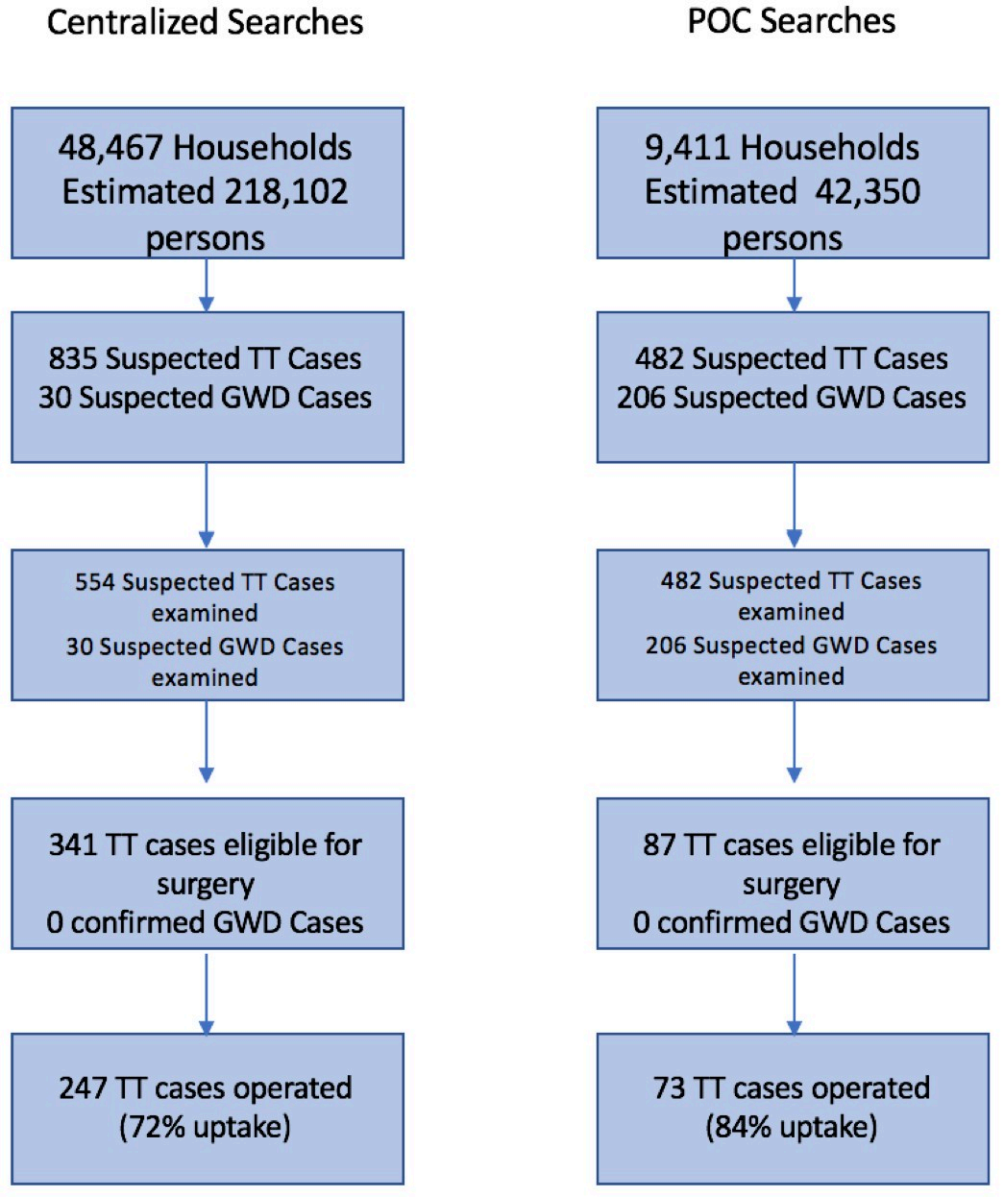
Flow chart contrasting results between methodologies.

The summarized results of both case search strategies are presented in Fig 2. Fig 3 shows the direct comparison of the outcome metrics between two case management approaches.

The centralized case management approach methodology reached 48,467 households, and an estimated 218,102 persons, using 959 volunteers and health workers (or about 480 teams) over a period of 34 days. The centralized case management approach also investigated a total of 30 individuals with signs or symptoms consistent with GWD. Additionally, 835 suspected TT cases were detected, for a rate of 17.2 TT cases suspected per 1,000 households reached. Of these, none were confirmed to be GWD and 554 TT suspected cases accepted examination (66.3% of cases found), of which 341 were eligible for surgery. The number of persons who accepted and presented for surgery was 247, resulting in an overall TT surgical uptake of 72.4% (247/341). The overall surgical refusal proportion was 27.6%. The TT surgical uptake percentage ranged from 58.5%–89.4% across the four districts using the centralized case management searches. (Fig 2).

The POC case management approach reached 9,411 households, and an estimated 42,350 persons, using 1,833 volunteers and health workers (or about 611 teams) over a period of 35 days. The POC searches investigated 206 individuals with signs or symptoms consistent with GWD. Furthermore, 482 TT cases were detected, for a rate of 51.2 cases of TT investigated per 1,000 households reached. Of these, none were confirmed to be GWD, and all 482 TT suspected cases accepted examination of which 87 were eligible for surgery. The number of persons who accepted and presented for surgery was 73, resulting in a surgical uptake of 83.9% (73/87). (Fig 2).

The total cost of the centralized searches was $11,550 (excluding surgical interventions) or $0.24 per household visited and $20.85 per TT case examined. The total cost for the POC searches was $9,626 (excluding surgical interventions) or $1.02 per household visited and $19.97 per TT case examined.

## Discussion

Both case management approaches resulted in successful investigation of suspected TT and GWD cases, and achieved high TT surgical uptake, demonstrating that it is possible to a) conduct integrated case searches for multiple diseases, and b) deliver TT surgery at the point of care and achieve high TT examination acceptance and TT surgery uptake. In the both methods, the surgical uptake was high, 72.4% overall in the centralized case management approach and 83.9% overall in the POC case management approach. Notably, while TT examination acceptance was high in the centralized case management approach (41% to 100%, with an average of 66.3%), the acceptance was 100% in the POC searches. This likely resulted from POC approach removing the time investment and logistical barriers to undergo examination as patients received examination and care at a location convenient to them immediately. [12–14] Additionally, an increase in the number of volunteers likely improved TT examination uptake as well. Several previous studies have indicated that the biggest barriers to TT examination and surgical uptake are time lost to travel, cost of travel, and time spent on travel and recovery. [11–13] For many of the world’s poor who live in rural areas, traveling to a central location for examination and surgery is a large investment—time spent traveling, waiting, and recovering equates to time not spent working, which creates an economic strain on the patient. The ability to deliver examination and surgery at the point of care removed these barriers by conducting examinations within or near patients’ homes, and doctors performing the surgery in a patient’s home. The results illustrate that offering TT examination and surgery at the point of care is not only feasible, but also potentially improves programmatic outcomes in regard to TT examination and surgical uptake.

In terms of cost, the centralized case management approach cost less on a per household basis ($0.24 per household visited) than the cost of the POC case management approach ($1.02 per household visited). The centralized case management approach was also more effective at reaching households and persons compared to the POC case management (48,467 households, and an estimated 218,102 persons, compared with 9,411 households, and an estimated 42,350 persons). However, the rate of TT cases investigated per 1,000 households visited was higher in the POC case management approach (50 cases of TT investigated per 1,000 households reached) when compared to the centralized case management approach (17 cases of TT investigated per 1000 households reached). This rate would be affected by the baseline prevalence of TT, but this should not be the sole metric to evaluate if house to house case searches should be implemented. Rather, this, in combination with the high number of households screened exhaustively, and the ability to offer surgical and treatment services at the point of care, suggest that such house-to-house searches can be implemented efficiently. Another advantage of this approach is that it can allow program decision makers to identify and document individuals who require morbidity management at a fine-scale and to inform treatment decisions to implement azithromycin mass drug administration. This will also prevent bias from oversampling TT patients who may be home versus healthy adults who may not be counted in standard monitoring surveys. Furthermore, in terms of cost per TT case examined, the POC case management approach ($19.97 per case examined) cost slightly less than the centralized case management approach ($20.85 per case examined), since 100% of the TT cases investigated also accepted examination; likely due to the reasons stated previously.

There are limitations. Firstly, because this report and evaluation is observational in nature, and was not a formal trial, we cannot directly compare which approach is the most effective. Second, cultural and religious differences between the search regions could have also have contributed to differential examination acceptance rate. We also lacked data on the underlying levels of TT in these districts, which prevented us from gaging the direct impact of the searches on TT prevalence in these areas. Finally, we lacked previous data on TT surgical uptake rates in Ghana and could not make a formal comparison as to how the current uptake compared to previous levels of surgical uptake. However, our aim here is not to evaluate the impact of the case search approach on TT prevalence, but rather to report and illustrate two ways of implementing case search approaches for TT, note the low cost and high TT examination acceptance and surgical uptake, and highlight the potential for integration with other NTD control and elimination programs.

The house-to-house case management approach offering examination and surgery at POC can be a workable and potent tool to meet eradication and elimination thresholds and help determine and relieve the burden of other NTDs. Many criticisms of point of care strategies stem from their relatively high cost when compared to centralized approaches. However, the fact that both methodologies presented here cost less than $12,000, reached tens of thousands of households, and achieved high TT surgical uptake, illustrates that integrated case searches using both methodologies are workable in the field. A POC case management approach might have been expected to cost more than a centralized case management approach due to the intensive logistical requirements. However, we demonstrate here that offering POC examination and surgery is feasible in a low-income setting, achieves better services utilization, and costs slightly less relative to the centralized approach. The integrated house-to-house search and POC service approach could also be used to identify and treat unreported patients in need of care, such as the elderly, infirmed, and especially those afflicted with lymphatic filariasis and leprosy. This approach would be effective for many other infectious disease and chronic disease elimination programs that require finding and treating cases; particularly in instances where afflicted persons cannot travel to a health center and would be missed in a passive referral system. The mixture of improved programmatic outcomes at similar or lower cost relative to a centralized approach warrants further exploration and usage of active case searches and POC approaches in low income settings for NTDs and other public health problems.
